# Risperidone-Induced Sexual Dysfunction Case Report

**DOI:** 10.7759/cureus.35357

**Published:** 2023-02-23

**Authors:** Gregory J Cichon, Syed F Qadri

**Affiliations:** 1 Psychiatry, Creighton University School of Medicine, Omaha, USA

**Keywords:** case report, antipsychotic, lurasidone, sexual dysfunction, risperidone

## Abstract

Treatment-emergent sexual dysfunctions are a common and distressing adverse effect of antipsychotic medication, particularly risperidone, which can result in medication noncompliance among patients with psychiatric disorders. In this case report, we present a 55-year-old male patient with a history of bipolar disorder type 1 who was admitted to an inpatient psychiatric facility due to a severe manic episode with psychotic features. The patient reported having previously taken risperidone for his bipolar symptoms but stopped taking it two months prior to hospitalization due to sexual side effects, including reduced libido and anejaculation. Comprehensive medical and psychiatric evaluations were performed during the patient's hospitalization, and his symptoms were treated with lurasidone instead of resuming risperidone. The patient's symptoms improved, and he was discharged with close outpatient follow-up for six months without symptoms of sexual dysfunction, depressive or manic symptoms, or adverse medication effects. This case adds to the growing body of literature on the adverse effects of risperidone, which is known to stimulate prolactin and contribute to sexual dysfunction in as many as 50-70% of patients, and highlights the potential benefits of switching to lurasidone, which has limited literature but as of this publication has not been associated with sexual dysfunction in clinical trials. However, more research is needed to fully understand the impact of antipsychotic switching on sexual side effects and the reluctance of patients to switch medications due to discomfort discussing these sensitive issues.

## Introduction

Sexual dysfunction is a prevalent side effect of antipsychotic medications, including risperidone, and affects approximately two-thirds of men and women after one year of use [1]. Risperidone is one such antipsychotic with high reported rates of sexual dysfunction [2]. Sexual dysfunction is the most frequently reported side effect of risperidone and the most cited reason for noncompliance among patients [3,4]. In men, risperidone can cause sexual dysfunction such as erectile dysfunction, decreased libido, and retrograde ejaculation. In women, it may cause decreased libido, vaginal lubrication, and orgasmic difficulty [1]. These side effects can be distressing for patients and can impact their quality of life.

Supplementary treatments, such as sildenafil, can be used to treat some of the sexual dysfunctions associated with risperidone [5]. However, its efficacy in treating sexual dysfunction caused by risperidone specifically has not been extensively studied, and more research is needed to determine its effectiveness.

Despite having such a large impact on quality of life, sexual dysfunction is often underreported by patients and underinvestigated by clinicians, resulting in medication noncompliance and worse outcomes. It is important to determine effective antipsychotic switching strategies and to keep in mind the sensitive nature of sexual dysfunction when asking about the adverse effects of these medications.

## Case presentation

Mr. W is a 55-year-old male with a history of bipolar disorder type 1 and anxiety who presented to our inpatient psychiatric facility due to a severe manic episode with psychotic features. The symptoms of mania started two months prior to hospitalization and gradually became worse. The symptoms include an irritable and agitated mood, a decreased need for sleep, an increased energy level, and grandiose thoughts. He also had psychomotor agitation with paranoid and delusional thoughts that people were out to get him and talking about him. He started acting on his mood and psychotic symptoms and destroyed property and engaged in a physical altercation. During a comprehensive psychiatric evaluation, the patient shared that he stopped taking his prescribed risperidone two months prior due to sexual dysfunction side effects, including reduced libido and anejaculation. He was still taking his other medications, which included amlodipine for hypertension, hydroxyzine as needed for anxiety, and nightly quetiapine for sleep and agitation. He had been following up with an outpatient psychiatric provider while experiencing these sexual side effects, though he was never asked about sexual function or whether he was comfortable sharing this information at the time. His symptoms of bipolar disorder were stable on risperidone 4 mg per day (2 mg of risperidone twice daily) and quetiapine 25 mg nightly, but due to the onset of sexual side effects, the patient decided to stop only the risperidone without informing his outpatient provider. He stated that the main reasons for discontinuing the medication were the sexual side effects, which he claimed resolved in two months after he stopped taking risperidone but started experiencing bipolar symptoms. He had previously taken risperidone during manic episodes many years prior for an unknown, brief duration but could not recall if he had any adverse effects at that time. He had also tried other antipsychotics such as quetiapine or aripiprazole previously, which were ineffective in controlling his symptoms. He also reported that during his current manic episode, he relapsed into using cocaine and cannabinoids off and on. He had not used cocaine for a few days prior to the onset of manic symptoms but relapsed due to his manic symptoms. He reported normal sexual function while off risperidone but on cocaine, so cocaine-induced sexual dysfunction was considered less likely. Still, his chronic cocaine use remained a consideration for his sexual dysfunction, as it has been shown to cause delayed ejaculation in men [6]. The patient was a reliable historian and collateral information was obtained to confirm his story. 

During hospitalization, his complete blood count, liver function test, renal function test, and thyroid function test were found to be within normal limits. A urine drug screen at the time of admission tested positive for cocaine and cannabinoids. A prolactin level was not performed since the patient was off risperidone or any other prolactin-elevating medications and the patient was not actively experiencing sexual side effects or symptoms of hyperprolactinemia, such as galactorrhea. A penile doppler flow was not performed based on the lack of symptoms of peripheral vascular disease on the physical exam and the prior medical workup. He was treated for bipolar symptoms during his inpatient hospitalization. The decision was made to switch to 20 mg of lurasidone daily instead of resuming risperidone based on recommended dosing strategies for bipolar disorder. The dose was titrated to 40 mg daily, and his symptoms of bipolar improved. At the same time, he was started on gabapentin (200 mg in the morning and 400 mg at night for comorbid back pain). The patient’s mood improved, and he started sleeping well with no anger outbursts or agitation. His symptoms of psychosis resolved as his mood symptoms improved. After stabilization and resolution of the patient’s presenting symptoms, he was discharged back to the community with the recommendation of outpatient follow-ups from our facility. He was followed as an outpatient at our facility for any further sexual dysfunction, manic symptoms, or medication adverse effects, none of which had been reported after six months.

## Discussion

Risperidone is categorized as a prolactin-stimulating antipsychotic since it blocks the D2 receptors. In this class of antipsychotics, hyperprolactinemia is a primary contributor to the adverse effects of sexual dysfunction [7]. One study found that risperidone produced higher levels of prolactin than other atypical antipsychotics and that this increase in serum prolactin was dose-dependent (Table 1) [8]. Blockage of 5HT2A and 5HT2C receptors in the prefrontal cortex likely also contributes to the sexual adverse effects [7]. Compared to olanzapine, the rate of sexual dysfunction is significantly higher (odds ratio: 2.02, 95% confidence interval between 1.63 and 2.48). Among the side effects of risperidone, the most commonly reported sexual side effects include decreased libido (37.8%), erectile dysfunction (32.1%), and ejaculatory disorder (including anejaculation and retrograde ejaculation) (32.6%) [9].

**Table 1:** Non-observational studies examining sexual dysfunction with risperidone use.

Author date	Type of study	Data collected	Population	Study objective	Treatment group	Main outcomes
Bo et al. [14]	Double blinded RCT	26 weeks	N=374, males and females, diagnosis of schizophrenia or other psychotic disorder	Prolactin-related symptoms with risperidone	N = 125 reduction of 50% of initial optimal dose of risperidone at week 4; N = 120 reduction of 50% of initial optimal dose of risperidone at week 26; N = 129 risperidone maintenance	Increased sexual dysfunction with increasing risperidone dose (P = 0.00)
Knegtering et al. [15]	Open label randomized	6 weeks	N=49, males and females, diagnosis of schizophrenia or other psychotic disorder	Sexual dysfunction with quetiapine vs. risperidone	N = 25 quetiapine; N = 24 risperidone	Reduced sexual dysfunction with quetiapine compared to risperidone (16% vs. 50%) (P = 0.00)
Ciudad et al. [16]	Open label randomized	1 year	N=247, males and females, diagnosis of schizophrenia or other psychotic disorder	Safety and tolerability of olanzapine vs. risperidone	N = 123 risperidone; N = 124 olanzapine	Patients on risperidone reported significantly more sexual dysfunction than patients taking olanzapine (21.1% vs. 7.3%; P = 0.00)
Knegtering et al. [17]	Open label randomized	6 weeks	N=27, males and females, diagnosis of schizophrenia or other psychotic disorder	Sexual dysfunction with aripiprazole vs. risperidone	N = 12 aripiprazole; N = 15 risperidone	Higher sexual dysfunction in risperidone group compared to aripiprazole (P = 0.00)
Tran et al. [18]	Double blinded RCT	28 weeks	N=339, males, diagnosis of schizophrenia or other psychotic disorder	Sexual dysfunction with olanzapine vs. risperidone	N = 172 olanzapine; N = 167 risperidone	Olanzapine was associated with lower sexual dysfunction compared to risperidone (P = 0.02)
Kinon et al. [19]	Open label randomized	4 months	N=54, males and females, diagnosis of schizophrenia or other psychotic disorder	Sexual dysfunction and prolactin after switch from 1^st^ generation antipsychotic or risperidone to olanzapine	N = 27 switch to olanzapine; N = 27 risperidone or 1st-generation antipsychotic maintenance	Significant improvement in overall scores of the Global Impression of Sexual Function for patients switching to olanzapine (P = 0.02)

Lurasidone, on the other hand, has not been associated with treatment-related sexual dysfunction in a recent clinical trial [10]. Lurasidone has a high binding affinity for D2, 5HT2A, and 5HT7 receptors, along with partial agonism of 5TH1A and no affinity for H1 or M1 receptors. Long-term treatment with lurasidone has not been shown to elevate prolactin levels [11]. These interactions may suggest lurasidone’s better tolerability profile and reduced sexual side effects compared to risperidone. One case study also showed a decrease in sexual dysfunction when switching to lurasidone from risperidone [12]. However, more research must be done to corroborate these findings, and we hope this case study adds to the body of literature in the area. We chose to switch from risperidone to lurasidone based on available clinical research and the patient’s prior inexperience with that particular antipsychotic.

A recent clinical trial was unable to produce antipsychotic switching strategies due to insufficient recruitment since many patients are reluctant to switch antipsychotics and sexual side effects are uncomfortable subjects to frequently discuss [13]. Until more data are available to effectively guide clinical decision-making, antipsychotic switching strategies for sexual adverse effects will be a challenge for both patients and clinicians to effectively address. We hope this case of risperidone-induced sexual dysfunction adds to the growing body of literature on sexual side effects causing medication noncompliance and one effective method of addressing it.

## Conclusions

Sexual dysfunction is a major contributor to noncompliance with antipsychotics, especially risperidone, as shown in this case. Clinicians should inquire regularly about these sexual side effects and consider alternative medications as the body of literature on this subject grows. Close monitoring of sexual side effects would not only prevent bipolar disorder relapse but it might also prevent patients from developing substance-use disorders.
